# Conditionally Replicative Adenovirus Controlled by the Stabilization System of AU-Rich Elements Containing mRNA

**DOI:** 10.3390/cancers12051205

**Published:** 2020-05-11

**Authors:** Yohei Mikawa, Mohammad Towfik Alam, Elora Hossain, Aya Yanagawa-Matsuda, Tetsuya Kitamura, Motoaki Yasuda, Umma Habiba, Ishraque Ahmed, Yoshimasa Kitagawa, Masanobu Shindoh, Fumihiro Higashino

**Affiliations:** 1 Department of Vascular Biology and Molecular Pathology, Faculty of Dental Medicine and Graduate School of Dental Medicine, Hokkaido University, Sapporo 060-8586, Japan; yoheimikawa@den.hokudai.ac.jp (Y.M.); towfik@den.hokudai.ac.jp (M.T.A.); ayayana9@den.hokudai.ac.jp (A.Y.-M.); tetsux@den.hokudai.ac.jp (T.K.); 2 Department of Oral Diagnosis and Medicine, Faculty of Dental Medicine and Graduate School of Dental Medicine, Hokkaido University, Sapporo 060-8586, Japan; ykitagaw@den.hokudai.ac.jp; 3 Department of Molecular Oncology, Faculty of Dental Medicine and Graduate School of Biomedical Science and Engineering, Hokkaido University, Sapporo 060-8586, Japan; elora@den.hokudai.ac.jp (E.H.); dr.ishraqueahmed@gmail.com (I.A.); 4 Department of Oral Molecular Microbiology, Faculty of Dental Medicine and Graduate School of Dental Medicine, Hokkaido University, Sapporo 060-8586, Japan; moyasuda@den.hokudai.ac.jp; 5 Department of Cancer Pathology, Faculty of Medicine, Hokkaido University, Sapporo 060-8638, Japan; habiba@med.hokudai.ac.jp; 6 Department of Nutrition, Faculty of Nursing and Nutrition, Tenshi College, Sapporo 065-0013, Japan; mshindoh118@gmail.com

**Keywords:** Oncolytic adenovirus, E1A, AU-rich element (ARE), HuR, ARE-mRNA, E1B55k, Hexon, *TNF-α*, *c-fos*, LPS

## Abstract

AU-rich elements (AREs) are RNA elements that enhance the rapid decay of mRNAs, including those of genes required for cell growth and proliferation. HuR, a member of the embryonic lethal abnormal vision (ELAV) family of RNA-binding proteins, is involved in the stabilization of ARE-mRNA. The level of HuR in the cytoplasm is up-regulated in most cancer cells, resulting in the stabilization of ARE-mRNA. We developed the adenoviruses AdARET and AdAREF, which include the ARE of *TNF-α* and *c-fos* genes in the 3′-untranslated regions of the E1A gene, respectively. The expression of the E1A protein was higher in cancer cells than in normal cells, and virus production and cytolytic activities were also higher in many types of cancer cells. The inhibition of ARE-mRNA stabilization resulted in a reduction in viral replication, demonstrating that the stabilization system was required for production of the virus. The growth of human tumors that formed in nude mice was inhibited by an intratumoral injection of AdARET and AdAREF. These results indicate that these viruses have potential as oncolytic adenoviruses in the vast majority of cancers in which ARE-mRNA is stabilized.

## 1. Introduction

The use of conditionally replicative adenoviruses (CRAd) is an attractive tool for cancer therapy. Many types of CRAd have been developed to date, some of which are currently undergoing clinical trials [1]. Two types of genetically engineered CRAd have been examined in detail [2]. The first group of viruses have mutations in the genes required for viral replication. For example, the E1A or E1B55k gene deleted-virus [3,4] has been effective at killing pRB- or p53-deficient cancer cells. The other group consists of viruses that possess a cancer-specific transcription system in the virus genes required for replication, such as E1A. For example, promoters of the telomerase gene [5] or prostate-specific antigen gene [6] are inserted into the 5′- untranslated region (UTR) of the E1A gene to produce CRAds that are specifically activated in cancer cells.

The control of mRNA decay is one of the important mechanisms of the gene expression system. AU-rich elements (AREs) are RNA elements commonly present in the 3′-UTR of certain mRNAs that encode many early response genes or growth-related genes such as proto-oncogenes, growth factors, and cytokines [7,8]. Multiple copies of the typical sequence AUUUA exist in ARE and target ARE-mRNA for rapid degradation [7,9].

HuR, a member of the embryonic lethal abnormal vision (ELAV) family of RNA-binding proteins, binds to ARE in order to protect ARE-mRNA from rapid degradation [9,10]. Although HuR is predominantly localized in the nucleus, it has the ability to shuttle between the nucleus and cytoplasm, and the stabilization of ARE-mRNA by HuR has been linked to its localization in the cytoplasm [10,11].

In cells transformed by the adenovirus oncogene product E4orf6, ARE-mRNA and its associated proteins, such as pp32 and HuR, are exported to the cytoplasm in a chromosome region maintenance 1 (CRM1)-independent manner [12]. E4orf6 may also stabilize ARE-mRNA, which endows it with the potential to transform cells [13,14]. The export of HuR and concurrent stabilization of ARE-mRNA do not solely depend on virus gene products and have been reported in many cancer cells. The cytoplasmic expression of HuR has been implicated in the malignancy of several types of carcinomas, such as colon cancer, and has also been suggested to contribute to the cancerous malignant phenotype [11]. Increased cytoplasmic HuR level was recently identified as an important prognostic marker in several cancers [15,16].

In the present study, we developed the oncolytic adenoviruses AdARET and AdAREF, possessing the ARE of the *TNF-α* and *c-fos* genes in the 3′-UTR of the *E1A* gene, respectively. The ability of these viruses to replicate was markedly higher in cancer cells than in normal cells and occurred in an E1A expression-dependent manner. These viruses exhibit cytolytic activity for cancer cells in vitro and in vivo. These findings indicate that the viruses have potential as oncolytic viruses. In the previous study, a virus with a COX-2 ARE in the 3′-UTR of E1A was developed [17]. This virus was developed primarily for cancer cells with ras mutations. AdARET and AdAREF were also effective in cancer cells that do not have the ras mutation. In addition, our virus also has reduced E1A expression, which means less damage to normal cells.

## 2. Results

### 2.1. Construction of an Adenovirus Including an ARE in the 3′-UTR of the E1A Gene and the Resulting Features of AdARET and AdAREF

**Figure 1:**
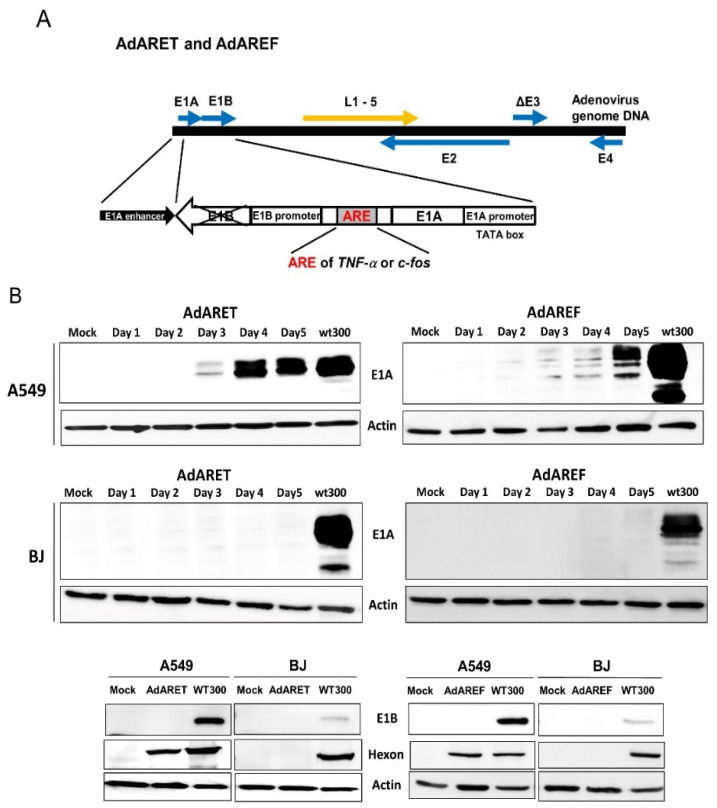
Structure of AU-rich element (ARE)-containing oncolytic adenoviruses and the expression of virus gene products. (**A**) Schematic representation of AdARET and AdAREF with the ARE of the *TNF-α* and *c-fos* genes in the 3′-UTR of the E1A gene, respectively. The location and direction of the inserted E1 region including ARE is indicated by a white arrow. Early (E1–4) and late (L1–5) genes are indicated by arrows. TATA box in E1A promoter is marked. (**B**) E1A expression in both new viruses (AdARET and AdAREF at a Multiplicity of Infection (MOI) 100 vp/cell day 1 to 5) and wild-type adenovirus (WT300 at an MOI 10 vp/cell, 24 h of infection) infected A549 and BJ cells were detected by western blot analysis. (upper and middle) E1B55k and hexon protein expression in the cells infected with the same viruses. (bottom) WT300 infected cells were used as a positive control, while non/mock infection was used as a negative control. β-actin expression was used as a loading control. The uncropped blots and molecular weight markers are shown in Figure S4.

In order to produce a new oncolytic adenovirus that replicates specifically in ARE-mRNA-stabilized cancer cells, we constructed an adenovirus including the ARE of the *TNF-α* and *c-fos* genes in the 3′-UTR of the E1A gene and designated them AdARET and AdAREF, respectively (Figure 1A). Since ARE-containing mRNA is degraded under normal conditions, but is stabilized in cancer cells, viral E1A expression was expected to be higher in cancer cells than in normal cells. We produced these viruses with the E1 region inserted in the opposite direction to create an oncolytic virus that is less harmful to normal cells. The transcriptional regulatory region of E1A of this virus is separated from the transcription initiation region including the TATA box by an enhancer, and transcription of the E1A gene is expected to be weaker and slower than in the cells infected with wild-type adenovirus. Additionally, these viruses fail to express E1B55k (although they can express E1B19k), as the E1 region, including the ARE, was inserted in the opposite direction and the E1B gene was interrupted.

As shown in Figure 1B, E1A protein expression was clearly detected in virus-infected A549 cells, but not in mock-infected A549 cells. The expression of E1A was absent in normal BJ cells, even if both viruses infected these cells, and E1B55k was not expressed in any cells, as expected. Usually, E1A protein expression starts 8 h after infection [18]; in the case of AdARET and AdAREF, it started slower than wild-type adenovirus (WT300) (Figure S1). On the other hand, infection with WT300 induced the expression of the E1A and E1B55k proteins in both cancer and normal cells (Figure 1B). We also estimated the expression of a hexon protein, which is translated from adenovirus late mRNA and is required to produce virus particles. The expression level of hexon was higher in cancer cells infected with both viruses than in normal cells, and correlated with the expression of the E1A protein (Figure 1B). These results indicate that, as expected, the E1A protein was expressed in high levels in cancer cells but at very low levels in normal cells. Additionally, virus late protein was expressed along with E1A expression in these cells, but the E1B55k protein was not expressed in either virus-infected cells.

### 2.2. Selective Replication of AdARET and AdAREF in Cancer Cells

**Figure 2:**
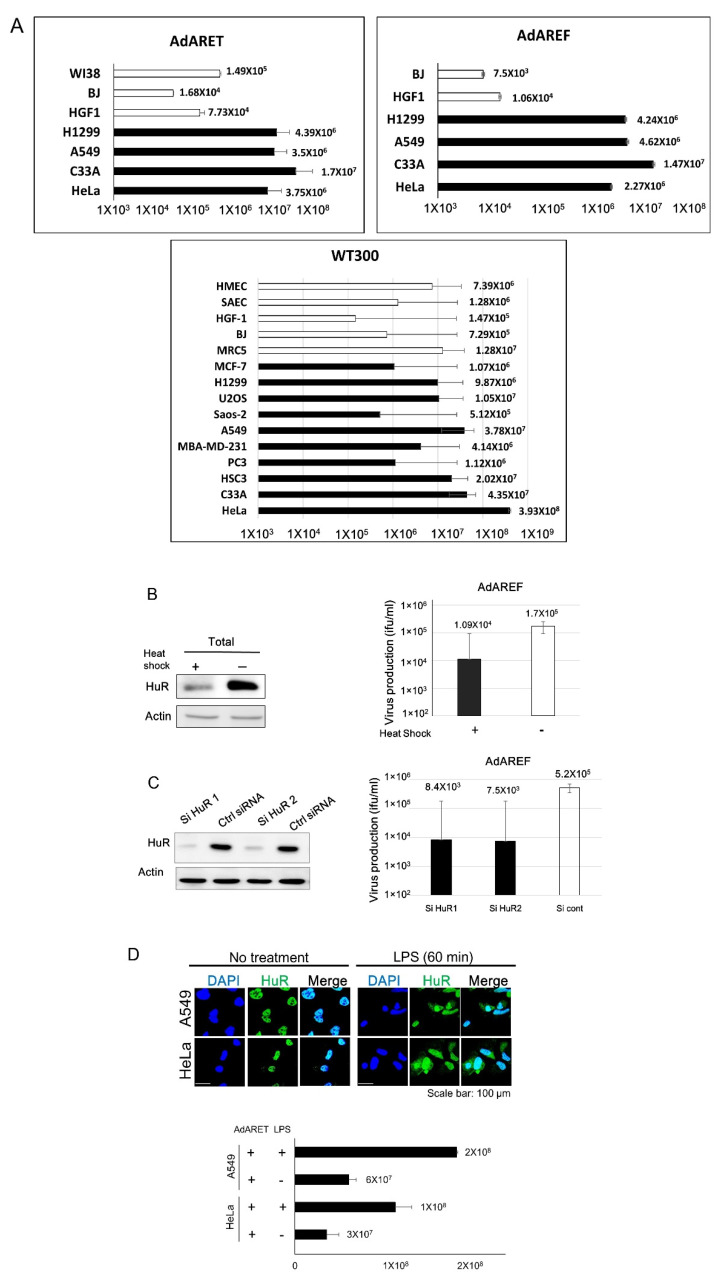
Productive efficiency and HuR dependence of AdARET and AdAREF. (**A**) Cancer (HeLa, H1299, A549, and C33A) and normal (BJ, HGF1, and WI38) cells were infected (72 h of infection) with AdARET and AdAREF at an MOI of 100 vp/cell, and the titer of the virus was determined by hexon staining in 293 cells. (upper) Cancer (MCF-7, H1299, U2OS, Saos-2, A549, MBA-MD-231, PC3, HSC3, C33A, and HeLa) and normal (HMEC, SAEC, HGF1, BJ, and MRC5) cells were infected (48 h of infection) with WT300 at an MOI of 10 ifu/cells, and titers were estimated. (lower) Each titer is indicated on the graph. (**B**) AdAREF-infected A549 cells were exposed to heat shock at 43 °C for 2 h and HuR expression was subsequently detected by western blot analysis. β-actin expression was used as loading control. (left) Each virus titer was estimated using the same method described in (**A**). (right) (**C**) A549 cells were transfected with an siRNA targeting HuR and a negative control siRNA and the expression of HuR was estimated by western blot analysis. (left) HuR KD-A549 cells were infected with AdAREF, and viral titers were determined as described in (**A**). (right) (**D**) The cytoplasmic HuR level was also estimated by confocal microscopy. HeLa and A549 cells were treated with LPS and then stained using an anti-HuR antibody, as described. In all cases, cell nuclei were visualized by staining with DAPI. Bars indicate 100 µm. Data shown are from a single experiment representative of three repeats giving similar results. (top) To assess the effect of LPS for viral replication, A549 and HeLa cells were treated with LPS and infected with AdARET, then the viral titers were determined as described in (**A**). Each titer (ifu/mL) is shown on the graph. Data are shown as the mean ± standard deviation of three independent experiments (bottom). The uncropped blots and molecular weight markers are shown in Figure S4.

In order to estimate the yields of AdARET and AdAREF, cancer cells (C33A, HeLa, H1299, and A549) and normal cells (BJ, HGF1, and WI38) were infected with these viruses, and virus titers generated after 72 h were detected by staining the viral hexon protein using 293 cells. In cancer cells, the production of AdARET was high, with titers ranging from 3.5 × 10^6^ to 1.7 × 10^7^ ifu/mL, while in the case of AdAREF, titers ranged from 2.27 × 10^6^ to 1.47 × 10^7^ ifu/mL. On the other hand, virus titers in normal cells were 2 to 4 logs lower than those in cancer cells. Additionally, the replication rates of WT300 were not so much different between cancer and normal cells (Figure 2A, Figure S2). These results indicated that AdARET and AdAREF selectively replicated in cancer cells.

### 2.3. Requirement of HuR for AdARET and AdAREF Replication

Since our developed viruses were expected to replicate in cancer cells through E1A-ARE mRNA stabilization, we determined whether the ARE-mRNA stabilization system was required for their replication. In order to estimate this, we examined the virus titer in heat shock (HS)-treated cancer cells, as this treatment down-regulates HuR, and HuR-targeted mRNAs were also decreased in HS-treated cells [19]. The HS treatment was expected to down-regulate the virus titer if AdAREF actually replicated using the ARE-mRNA stabilization system. As shown in Figure 2B, a 2-h HS treatment induced a reduction in the expression of the HuR protein in A549 cells, as expected. We then examined virus titers in the same HS-treated A549 cells. AdAREF-infected cells were treated with HS both immediately and 24 h after infection and virus titers were counted. The HS treatment significantly reduced production of the virus (Figure 2B).

As HS treatment has many influences on cells other than HuR degradation, we also performed HuR knockdown (KD) using siRNA to confirm that the observed effect was due to HuR-depletion effect. The virus replication following treatment with two HuR siRNA was much lower than that in control siRNA transfected cells (Figure 2C).

We understood that a HuR depletion suppressed the virus growth, thus, we subsequently examined contrary the effects of cytoplasmic HuR relocalization on the virus growth. In order to activate the cytoplasmic HuR relocalization, HeLa and A549 cells were treated with lipopolysaccharide (LPS) [20,21]. Immunostaining analysis showed that LPS induced HuR cytoplasmic exportation (Figure 2D). AdARET replication was obviously upregulated in these cells with enhanced HuR exportation (Figure 2D).

Taken together, these results indicate that HuR is required for AdARET and AdAREF replication and that the ARE-mRNA stabilization system is necessary for the replication of these viruses. Our previous report showed that HuR depletion (HS and HuR KD) causes the downregulation of the replication of WT300 because HuR is required for full replication of adenovirus [22]. However, since the downregulation rate of WT300 was lower than that of AdAREF, the dependency of HuR of AdAREF is significantly higher than that of WT300.

### 2.4. Cytolytic Potential of AdARET and AdAREF

**Figure 3:**
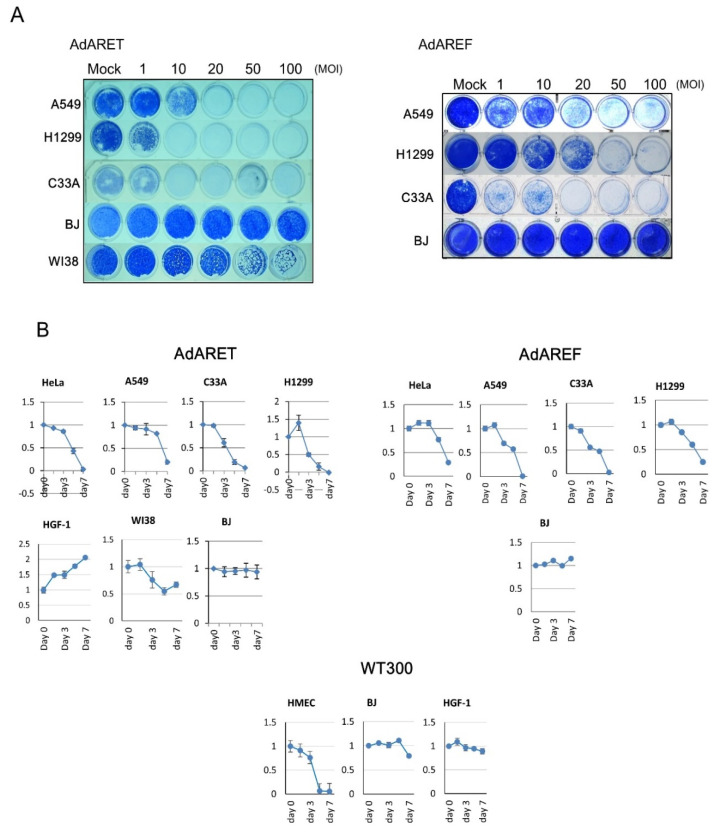
In vitro cell lysis activity of AdARET and AdAREF. (**A**) Cancer (A549, H1299, and C33A) and normal (BJ and WI38) cells were infected with AdARET and AdAREF at the MOIs indicated. Cells were stained with Coomassie brilliant blue seven days after infection. Living cells were stained blue. (**B**) The cell viability of AdARET, AdAREF, and WT300-infected cells was measured using the XTT assay. The indicated cancer and normal cells were infected with the virus at an MOI of 100 vp/cell and cell viabilities were estimated 0, 1, 3, 5, and 7 days after infection.

In order to assess the cell lysis activity mediated by AdARET and AdAREF, we examined the cytopathic effects (CPE) of infected cells. Three cancer cell lines (A549, H1299, and C33A) and normal cell lines (BJ and WI38) were infected with AdARET and AdAREF at Multiplicity of Infections (MOIs) of 1, 10, 20, 50, and 100. One week later, cytotoxicity was estimated by staining the remaining cells with Coomassie brilliant blue (Figure 3A). All cancer cells were killed by these viruses in a dose-dependent manner, whereas several cell lines, such as A549, survived infections with low MOIs. On the other hand, most of the normal cells survived all infections.

Cell viability was estimated using the XTT assay in an attempt to further explore the cell lytic potential of AdARET and AdAREF further (Figure 3B). Cancer cells and normal cells were infected with both viruses at MOI 100 (vp/cell) and cell viability was analyzed 0, 1, 3, 5, and 7 days after infection (Figure 3B). AdARET infection resulted in reductions in the viability of C33A and H1299 cells three days after infection, and most cancer cells died after seven days. AdAREF infected A549 and C33A cells showed low viabilities three days after infection, and almost all cancer cells were killed within one week. In contrast, the viability of normal cells was less affected by infection with these viruses than WT300 (Figure 3B). Taken together, these results indicate the *in vitro*-selective cytolytic activity of AdARET and AdAREF.

### 2.5. In Vivo Effects of AdARET and AdAREF

**Figure 4:**
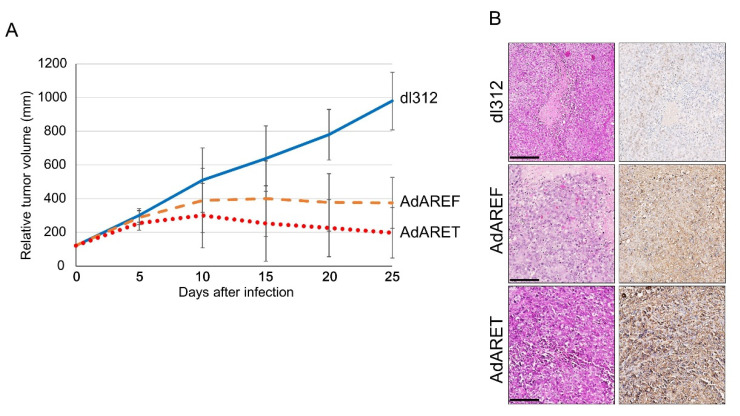
In vivo antitumor effects of intratumorally injected AdARET and AdAREF in HeLa xenograft nude mice. (**A**) HeLa cells were injected subcutaneously into nude mice to form a tumor with a diameter of approximately 5 mm (after approximately 3 weeks). 10^9^ vp (100 μL) of dl312, AdARET and AREF were injected intratumorally into the tumor on days 1 and 4. At least five mice were used for each group. Tumor volumes were measured twice a week and the results obtained are shown as the mean of relative volumes ± SD. (**B**) H&E and immunohistochemistry staining of HeLa S3 tumors treated with dl312, AdARET and AdAREF in deparaffinized section. Adenovirus detection (brown precipitation) was performed by immunostaining using anti-adenovirus antibody in all viruses injected tumors. Scale bar indicates 100 µm.

In order to examine the in vivo effects of AdARET and AdAREF using a tumor xenograft model, HeLa cells were implanted into the hind flanks of nude mice and the tumors that developed were injected twice with 10^9^ vp AdARET, AdAREF, or dl312, which is an E1A gene-deleted type5 adenovirus [23], as a control. The volumes of tumors injected with both viruses were significantly smaller than those injected with dl312 (Figure 4A). Two out of five tumors disappeared completely following the virus injection (data not shown). Additionally, we estimated the in vivo effects of both viruses using PBS as a control (Figure S3). Thus, AdARET and AdAREF exerted significant effects on human tumor xenografts in nude mice.

To investigate the effects of AdARET and AdAREF on tumors, we examined whether these viruses could replicate in the tumor. Immunohistochemical analysis was performed on tumors obtained after viral treatment. AdARET and AdAREF injected tumors showed abundant adenoviral protein expression compared to dl312 injected tumors. Detection of higher levels of viral protein in AdARET injected tumor tissues indicated that higher levels of viral replication (Figure 4B).

These data indicate that these viruses replicate in xenografted tumors to inhibit their growth, suggesting the in vivo efficacy of these viruses.

## 3. Discussion

In this study, we described the construction and features of AdARET and AdAREF, two new oncolytic adenoviruses. These viruses contain the ARE of the *TNF-α* and *c-fos* genes in the 3′-UTR of the E1A gene, which allows them to replicate specifically in ARE-mRNA-stabilized cancer cells. Indeed, E1A expression was high specifically in cancer cells and the productive rate of these viruses were very high in several cancer cells, but were very low or negligible in normal cells. These viruses exhibited selective cytolytic activity for cultured cancer cells in vitro. In the nude mouse xenograft model, infection with these viruses significantly reduced tumor volume. In order to produce a virus that would exert less damage to normal cells, the viruses were created with the E1 region inserted in the reverse direction. Furthermore, these viruses could not express the E1B55k gene due to its interruption. The expression of E1A protein from these viruses was lower and slower than in wild-type adenovirus infected-cells and the E1B55k protein was not visible by western blot analysis. These results indicate that AdARET and AdAREF have potential as oncolytic viruses. To the best of our knowledge, there are few reports describing oncolytic viruses with tumor selectivity based on the level of mRNA stability. As mentioned, AdARET and AdAREF failed to express E1B55k due to the truncation in this gene. This feature is the same with the E1B55k gene deleted-adenoviruses such as Onyx-015 or H101, which have already been applied clinically [3,24]

Since these viruses were expected to replicate in cancer cells through E1A-ARE mRNA stabilization, we determined whether the ARE-mRNA stabilization system was required for virus replication. In order to estimate this, we used three kinds of experiments. Two assays were performed to examine the down-regulation HuR expression and one experiment was used to observe the effect of the up-regulation of cytoplasmic HuR. We confirmed the virus titer in heat shock (HS)-treated cancer cells, as the amount of HuR was down-regulated, and HuR-targeted mRNA was also decreased in HS-treated cells [18]. This HS treatment would down-regulate the virus titer if AdAREF actually replicated using the ARE-mRNA stabilization system. The titer of AdAREF in HS-treated cells was lower than that of non-treated cells (Figure 2B). The reduction of AdAREF virus replication has also been seen with HuR-KD cells. HuR-KD, with two kinds of siRNA specific for HuR, reduced HuR expression and virus production (Figure 2C). Conversely, when HuR export was activated by LPS treatment, replication of AdARET increased (Figure 2D). These results indicate that the developed viruses grow in an ARE-mRNA stabilization system dependent manner. Since ARE-mRNA is usually known to be stabilized in many types of cancer cells [11], AdARET and AdAREF can be expected to be effective in numerous cancers.

In a previous study, an adenovirus including the ARE of COX-2 in the 3′-UTR of the E1A gene was shown to be a conditionally replicating virus [17]. This virus replicated selectively in RAS/P-MAPK-activated cancer cells because ARE-mRNA was stabilized under the activated RAS pathway. In the case of AdARET and AdAREF, cytolytic activity was not dependent on the RAS/P-MAPK pathway, because the virus exhibited oncolytic activity for HeLa cells, which carry a normal ras gene. These findings indicate that these viruses are effective for cancer cells carrying unmutated ras.

Since the stability of ARE-mRNA has been reported in many biological or pathological events, such as inflammation, viral infection, hypoxia, and UV irradiation, AdARET and AdAREF have potential in the treatment of diseases with these biological features.

## 4. Materials and Methods

### 4.1. Cell Lines and Antibodies

The human lung cancer cell lines A549 and H1299, cervical carcinoma cell lines HeLa, HeLa S3, and C33A, breast cancer cell lines MCF-7 and MDA-MB-231, osteosarcoma cell lines U2OS and Saos-2, prostate cancer cell line PC3, oral cancer cell line HSC-3, human embryonal kidney cell line (transformed by the adenovirus E1 gene) 293, human foreskin fibroblast cell line BJ, gingival fibroblast cell line HGF1, lung fibroblast MRC5, mammary epithelial cell line HMEC, and small airway epithelial cell line SAEC were all used in the present study. All cells were obtained from the American Type Culture Collection or Lonza and cultured in DMEM containing 10% FBS with antibiotics at 37 °C in a 5 -% CO_2_ atmosphere under humidified conditions.

For western blot analysis, the following antibodies were used: antibodies specific to E1A (M58, sc-58658, Santa Cruz Biotechnology, Dallas, TX, USA), E1B55k (2A6, generous gift from T. Shenk), HuR (3A2, sc-5261, Santa Cruz Biotechnology, Dallas, TX, USA), Hexon (Abcam, Cambridge, United Kingdom), β-actin (Actin(C4) hrp, sc-47778, Santa Cruz Biotechnology, Dallas, TX, USA), β-tubulin (05–661, EMD Millipore Corp., Darmstadt, Germany), Lamin B (C-20, sc-6216, Santa Cruz Biotechnology, Dallas, TX, USA), and Anti-Adenovirus Type 5 antibody (ab6982, Abcam, Cambridge, United Kingdom) as primary antibodies. Anti-mouse (m-IgGκ BP-HRP: sc-516102, Santa Cruz Biotechnology, Dallas, TX, USA) and Anti rabbit (mouse anti-rabbit IgG-HRP: sc-2357, Santa Cruz Biotechnology, Dallas, TX, USA) were used as secondary antibodies.

### 4.2. Construction of AdARET and AdAREF

AdARET and AdAREF were constructed using a pXhoIC plasmid [25] and pAxcwit2 cosmid (Takara Bio., Kusatsu, Shiga, Japan). pXhoIC contained the E1 region of the type 5 human adenovirus genome. A 40-base pair of synthesized ARE fragment (5′-gtgattattt attatttatt tattatttat ttatttacag-3′) from the *TNF-α* and a 69-base pair of synthesized ARE fragment (5′- ttttattgtg tttttaattt atttattaag atggattctc agatatttat atttttattt tattttttt -3′) from the *c-fos* genes were inserted into the HpaI site in the 3′-UTR of the E1A gene of pXhoIC. A fragment of the E1 region was then amplified by PCR, and inserted into the deleted E1 region of the adenovirus genome in the pAxcwit2 cosmid using SmiI-restricted endonuclease. The entire genome of AdARET and AdAREF were isolated by cutting using PacI and each fragment was then transfected into 293 cells. Virus particles were concentrated by several rounds of viral infection and cells were collected in order to prepare a virus lysate by subjecting them to three cycles of freezing and thawing. The titers of the infectious unit (ifu) of these viruses were determined using the Adeno-X^TM^ Rapid Titer Kit (Cat no-632250, Clontech Laboratories, Mountain View, CA, USA) and 293 cells according to the manufacturer’s instructions. In order for a virus to be used in in vivo experiments, its extract was purified using a Fast Trap adenovirus purification and concentration kit (MILLIPORE) according to the manufacturer’s protocols. A Quick titer adenovirus quantitation kit (VPK-106, Cell Biolabs, San Diego, CA, USA) was used to count virus particles.

### 4.3. Western Blot Analysis

Western blot analysis was performed as previously described [26]. Cells were lysed with RIPA buffer (150 mM NaCl; 25 mM Tris-HCl, pH 7.6; 1% Nonidet P-40; 1% sodium deoxycholate; 0.1% SDS) containing protease inhibitors. Equal amounts (20 µg) of total protein were separated by 10% sodium dodecyl sulfate-polyacrylamide gel electrophoresis (SDS-PAGE) and transferred onto polyvinylidene difluoride membranes (Millipore, Billerica, MA, USA). The binding of antibodies was visualized using Supersignal West Femto Maximum Sensitivity Substrate (Cat-34095, Thermo Fisher Scientific, Waltham, MA, USA).

### 4.4. In Vitro Virus Proliferation Assay

Human tumor and normal cells were seeded at 5.0 × 10^4^ cells/well 24 h before infection. Cells were infected with AdARET and AdAREF at an MOI of 100 vp/cell. These infected cells were incubated at 37 °C for 72 h, then collected, and a virus lysate was prepared as described above. Titers of the viruses were determined using the Adeno-XTM Rapid Titer Kit (Clontech, Laboratories, Mountain View, CA, USA) and 293 cells.

### 4.5. HuR Manipulation

For HuR knockdown, Lipofectamine RNAiMAX (Invitrogen; Thermo Fisher Scientific, Waltham, MA, USA) was used to transfect HeLa cells with 20 nM each of siRNAs targeting HuR 1 (5′-AAG UGC AAA GGG UUU GGC UUU UU-3′) or HuR 2 (5′-AAU CUU AAG UUU CGU AAG UUA UU-3′) or a negative control siRNA (5′-TCT TAA TCG CGT ATA AGG CTT-3′; Qiagen, Hilden, Germany). Forty-eight hours after transfection, A549 cells were infected with AdARET and AdAREF. After 48 h of infection, all cells were scrapped to purify total protein, and the virus lysate was prepared with three freeze-thaw cycles. Viral titers were detected by using the Adeno-X Rapid Titer kit (Clontech Laboratories, Laboratories, Mountain View, CA, USA) and 293 cells.

For heat shock treatment, A549 cells were kept at 43 °C for 2 h immediately after being infected with AdARET and AdAREF. Cells were heat shocked (for 2 h) again 24 h after infection. Cells were harvested at 24 h after infection and the viral titers were determined as described above.

In order to promote cytoplasmic localization of HuR, cells were treated with 25 ng/mL LPS (E. Coli 0111: B4, Sigma-Aldrich, St Louis, MO, USA).

### 4.6. Immunocytochemistry

A549 and HeLa cells were grown over coverslips in 24 well plates at 60–80% confluence and treated with LPS (25 ng/mL). For HuR staining, cells were rinsed twice with phosphate buffered saline (PBS), fixed for 10 min at RT with 4% paraformaldehyde in PBS at room temperature, blocking and permeabilization was completed using 1% BSA plus 0.25% Triton X-100 in PBS at room temperature. The fixed cells were subsequently incubated with HuR primary antibody for overnight at 4 °C followed by Alexa Fluor 488 secondary antibodies for 1 h at room temperature, respectively. DAPI was used to counterstain cell nuclei before cells were mounted on slides using Mountant permafluor (Thermo scientific, FM 111212A, Waltham, MA, USA). Cells were observed with an IX71 inverted microscope (Olympus, Tokyo, Japan). Image acquisition was performed with the Olympus Fluoview Software (FV10-ASW 4.2 viewer).

### 4.7. Cytopathic Effect Assay and Cell Viability Assay

Human cancer and normal cells were plated on 24-well plates (5 × 10^4^ cells/well). Twenty-four hours later, cells were infected with AdARET and AdAREF at a multiplicity of infection (MOI) of 1, 10, 20, 50, or 100 virus particles (vp)/cell and maintained for an additional seven days. Cells were then fixed and stained with Coomassie brilliant blue. A 2–3-bis [2-methoxy-4-nitro- 5-sulfophenyl]-2H-tetrazolium-5-carboxanilide inner salt assay was used to examine cell viability. Cells were seeded on 96-well plates at a density of 3.0 × 10^3^ cells/well 24 h before viral infection. Twenty-four hours later, AdARET and AdAREF were infected at a MOI of 100 vp/cell. Cell viability was determined using an XTT assay on days 3 and 7 with Cell proliferation kit II (Cat no-11465015001, Roche Diagnostics, Roche, Germany) according to the manufacturer’s protocol.

### 4.8. In Vivo Human Tumor Model

Human Cervical Cancer HeLa S3 cells (1.0 × 106 cells/mouse) were injected subcutaneously into the flanks of female BALB/cAJc1-nu/nu nude mice (5-week-old and 20–22 g) and permitted to grow to approximately 9–10 mm in diameter. Mice were randomly divided into three groups (five per group), and 10^9^ vp (100 µL) of dl312, AdAREF and AdARET were injected twice (at days 1 and 4) directly into the tumor. The perpendicular diameters of the HeLa S3 tumors were measured every four or five days. Tumor volumes were calculated using the following equation: volume (mm^3^) = A × B^2^ × 0.5 (where A is the longest diameter and B is the shortest diameter). The body weight and motor activity of each animal was monitored as indicators of general health and toxicity. The mice were sacrificed by cervical dislocation 25 days following injection of the virus. All techniques involving animals in this study were performed according to the ethical standards of the Animal Care and Use Committee of the Hokkaido University. Sapporo, Japan (Permission number for the animal experiment: 19–0099).

### 4.9. Immunohistochemistry

Immunohistochemical staining was performed with serial tissue sections (4.5 µm thick) from formalin-fixed, paraffin-embedded tissue blocks. In brief, all sections were deparaffinized in xylene, rehydrated in graded alcohol, and subjected to antigen retrieval by heat treatment in Tris-ethylenediaminetetraacetic acid (TE) buffer. In order to inhibit endogenous peroxidase activity, the slides were then immersed in 3% hydrogen peroxide (Sigma-Aldrich, St. Louis, MO, USA) for 5 min, and then blocking solution [1% bovine serum albumin (Sigma-Aldrich) in phosphate-buffered saline (PBS)] for 30 min. The immunohistochemical detection of Adenovirus 5 (Abcam, ab6982) was performed using anti-Adenovirus Type 5 (Rabbit Adenovirus antibody, dilution, 1:1,000; catalog no., ab6982; Abcam) in PBS in a humidified chamber at 4 °C overnight. The sections were then subjected to anti-rabbit secondary antibody (H1901, Nichirei Bioscience, Tokyo, Japan) at 37 °C for 30 min, followed by antibody detection using a peroxidase-conjugated streptavidin-diaminobenzidine (DAB) readout system (DAKO), and counterstaining with DAPI. Rinses were carefully performed with several changes of PBS between each stage of the procedure (5 min washes repeated three times). Images were randomly captured using a nanozoomer slide scanner and NDPViewer (NanoZoomer 2.0 HT, version 2.3.27, Hamamatsu, Shizuoka, Japan).

## 5. Conclusions

In this study, we developed the CRAds AdARET and AdAREF, which contained the ARE of the *TNF-α* and *c-fos* genes in the 3′-UTR of the E1A gene, respectively. The expression levels of E1A of these viruses in cancer cells were markedly higher than in normal cells and resulted in cancer cell specific replication. The replication of these viruses depended upon the HuR, which is required for the stabilization of ARE-mRNA. These viruses exhibit cytolytic activity for cancer cells in vitro and in vivo. These findings indicate that these viruses have potential as an oncolytic virus.

## Supplementary Materials

The supplementary figures are available online at https://www.mdpi.com/2072-6694/12/5/1205/s1, Figure S1: Expression of E1A protein in AdARET and WT300 infected A549 cells, Figure S2: Comparison of replications of AdARET, AdAREF and WT300, Figure S3: In vivo antitumor effects of intratumorally injected AdARET in HeLa S3 xenograft nude mice, Figure S4: The uncropped blots and molecular weight markers of Figure 1B and Figure 2B,C.
