# Supplementary material for: Conditionally Replicative Adenovirus Controlled by the Stabilization System of AU-Rich Elements Containing mRNA
Source: Cancers (Basel). 2020 May 11;12(5):1205. doi: 10.3390/cancers12051205 (PMC7281395; doi:10.3390/cancers12051205)
Supplement: Supplementary file 1 [file cancers-12-01205-s001.pdf]

Supplementary Material

Conditionally Replicative Adenovirus Controlled by the Stabilization System of AU-Rich Elements Containing mRNA

Yohei Mikawa, Mohammad Towfik Alam, Elora Hossain, Aya Yanagawa-Matsuda, Tetsuya Kitamura, Motoaki Yasuda, Umma Habiba, Ishraque Ahmed, Yoshimasa Kitagawa, Masanobu Shindoh and Fumihiro Higashino *

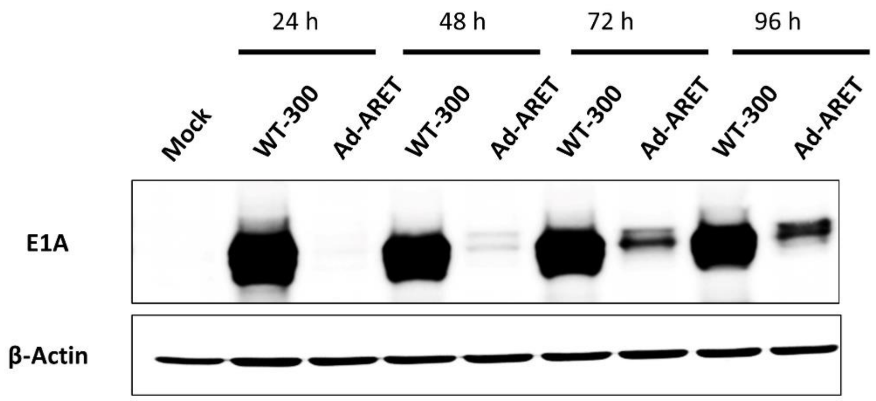

Figure S1. Expression of E1A protein in AdARET and WT300 infected A549 cells. Both viruses were infected with A549 cells for 24, 48, 72 and 96 hrs. Total cell extracts were prepared, then the expression of E1A and β-actin was estimated by western blot analysis.

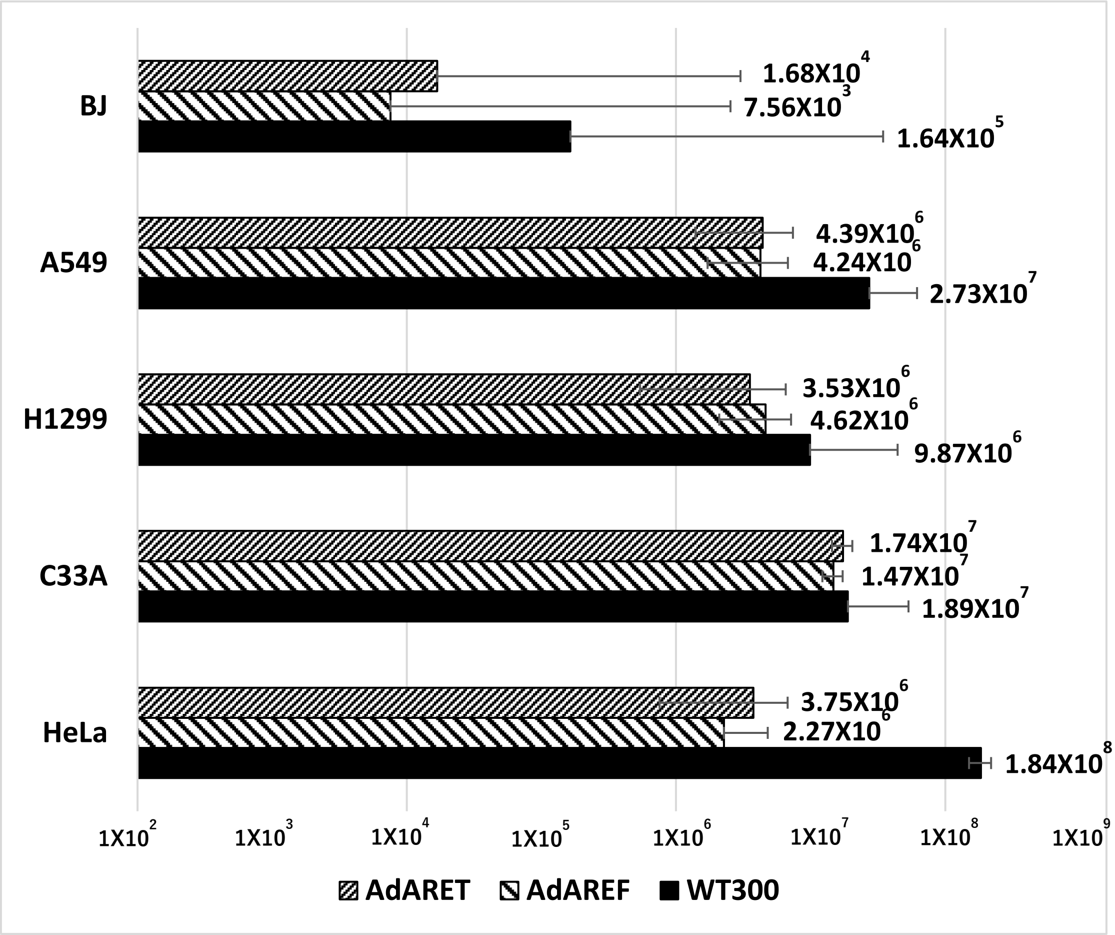

Figure 2S. Comparison of replications of AdARET, AdAREF and WT300. The data of Figure 2A were incorporated to compare the replication rate of each virus.

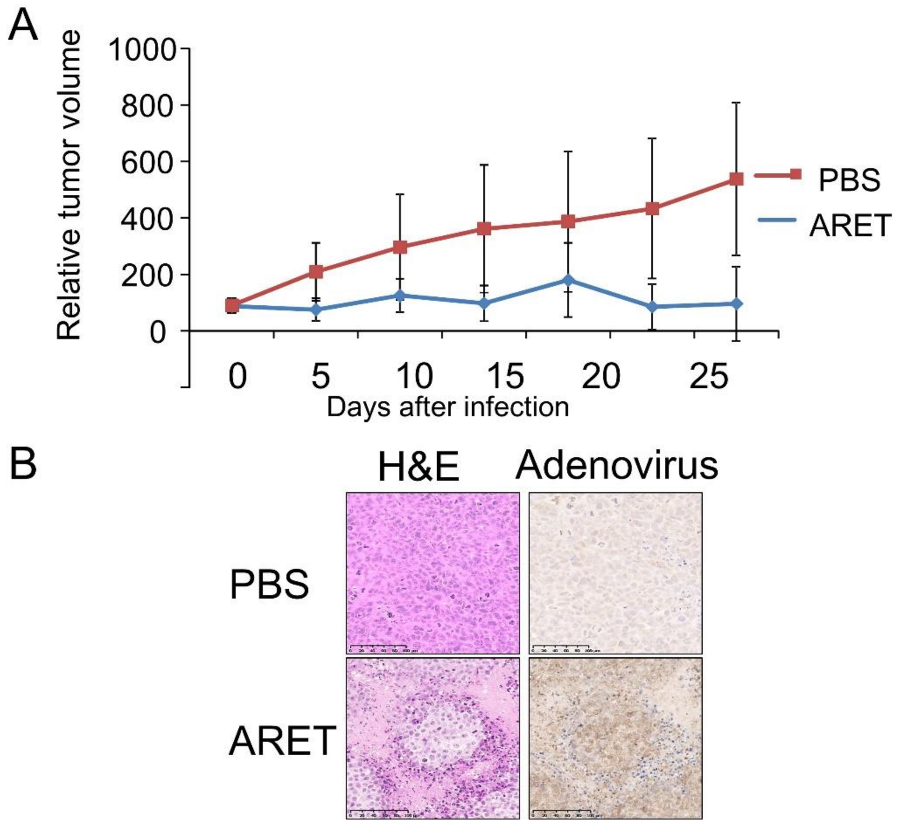

Figure 3S. *In vivo* antitumor effects of intratumorally injected AdARET in HeLa S3 xenograft nude mice. (A) HeLa S3 cells were injected subcutaneously into nude mice to form a tumor with a diameter of approximately 5 mm (after approximately 3 weeks). 10^9 vp (100 μl) of AdARET and PBS were injected intratumorally into the tumor on days 1 and 4. At least five mice were used for each group. Tumor volumes were measured twice a week and the results obtained are shown as the mean of relative volumes ± SD. (B) H&E and immunohistochemistry staining of HeLa S3 tumors treated with PBS and AdARET in deparaffinized section. Adenovirus detection (brown precipitation) was performed by immunostaining using anti-adenovirus antibody in all viruses injected tumors. Scale bar indicates 100 μm.

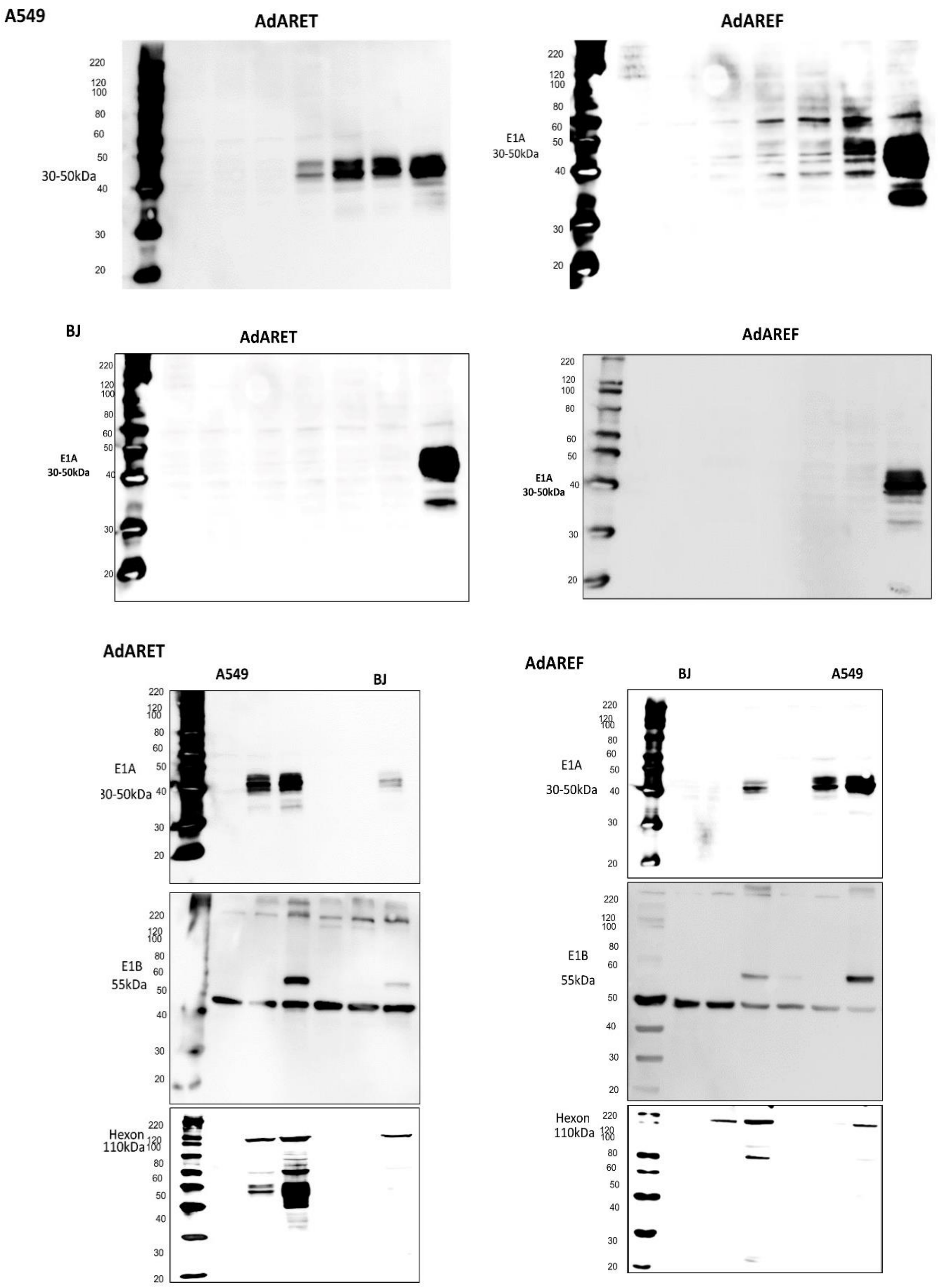
A549
AdARET
AdAREF
30-50kDa
E1A
30-50kDa
BJ
AdARET
AdAREF
E1A
30-50kDa
E1A
30-50kDa
AdARET
A549
BJ
E1A
30-50kDa
E1B
55kDa
Hexon
110kDa
AdAREF
BJ
A549
E1A
30-50kDa
E1B
55kDa
Hexon
110kDa
220
120
100
80
60
50
40
30
20

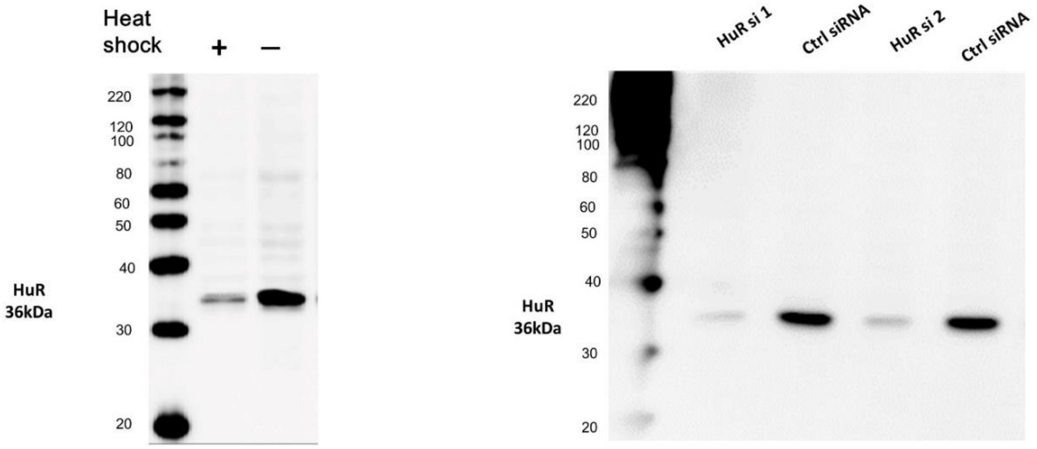

Figure 4S. Uncropped blots and molecular weight marker for Figure 1B, 2B and 2C.

© 2020 by the authors. Licensee MDPI, Basel, Switzerland. This article is an open access article distributed under the terms and conditions of the Creative Commons Attribution (CC BY) license (http://creativecommons.org/licenses/by/4.0/).
